# The Rise and Fall of the P. M. B. A.

**Published:** 1889-08

**Authors:** 


### THE RISE AND FALL OF THE P. M. B. A. OF TEXAS.

| | | | | |
|---|---|---|---|---|
| Assessment | No. 1 | netted | Mrs. W. H. Park | $55 00 |
| " | No. 2 | " | Mrs. D. Sayres | 109 00 |
| " | No. 3 | " | Mrs. Albert Welch | 120 00 |
| " | No. 4 | " | Mrs. A. R. Kilpatrick | 143 70 |
| " | No. 5 | " | Mrs. S. F. Starley | 135 50 |
| " | No. 6 | " | Mrs. Wm. Glover | 121 00 |
| " | No. 7 | " | Mrs. Y. D. Harrington | 120 00 |
| " | No. 8 | " | Mrs. F. A. Thompkins | 106 00 |
| " | No. 9 | " | Miss Minnie Buck† | 79 00 |
| " | No. 10 | " | Dr. W. G. Jamison* | 73 00 |
| " | No. 11 | " | Mrs. F. E. Maddox* | 73 00 |
| " | No. 12 | " | Mrs. J. M. Ross* | 59 00 |
| Total disbursed | | | | $1,194 20 |
| Average to each widow | | | | $99.50 |

The following is the list of members who paid assessment No. 12, benefit of Mrs. J. M. Ross:

Drs. Daniel, Burts, Sholars, Cupples, Swearingen, Day, Duchiene, Dupree, Towsey, Matthews, Styles, Sterrett, Myers, Powell, Hardy, Weller, Paulus, Ball, Tidwell, Throckmorton, Hons, Perkins, Ford, Litten, Thorpe, Fox, Martin, Gregg, Field, Wil-

*Receipt published below.
†Receipt in June number.

son, Evans, Wadgyman, Fennell, Shuford, Burke, Bowers, Patten, Patten, Lancaster, Watlington, Fry, Kaiser, Bell, McCreary, Witherspoon, Jones, Styles, Barbee, Nicholson, Starnes, Harmer, Burroughs, Seymour; Hewsen, Atkinson, Rape, Hill, Wailes, Crawford, Getzwiller, Wolff, Music, Brown, Dupre—64.

Sixty-four dollars, less expense of collecting, as follows:

| | |
|---|---|
| Postal cards and printing . . . . . . . | $2 75 |
| Second notices and receipts postage . . . | 2 00 |
| Exchange . . . . . : . . . . . | 25 |
| | $5 00 |
| Net . . . . . . . . | $59 00 |

The above members (except three) and the following, paid Nos. 9, 10 and 11, to-wit: Drs. Coffey, Webb, Reid, Ross (J. M.), Osborn, Q. C. Smith, Richmond, Taylor, (M. A.), Talley, Bass, Harrison, sen., Harrison, jr., Grace, Sams—78.

Seventy-eight dollars, less expense, $5—$73.

[In Miss Buck's case there was one dollar more (Dr. Beck) and no deduction was made for expense of collecting.]

$73.00. RUSK, TEXAS, July 27, 1889.

Received of Dr. W. A. Morris, President, and Dr. F. E. Daniel, Secretary, seventy-three dollars, the net amount collected under assessment No. 11 P. M. B. A., for benefit of certificate held by the late Dr. J. T. Y. Jamison. W. G. JAMISON.

$73.00. CORSICANA, Texas, July 22, 1889.

Received of Dr. W. A. Morris, President, and Dr. F. E. Daniel, Sec. and Treas. P. M. B. A. of Texas, seventy-three dollars, the net proceeds of assessment No. 10 for benefit of widow of Dr. L. T. Maddox, of Birdston.

S. G. MULLINS,
for MRS. FLORA E. MADDOX.

$59.00. OFFICE PHYS. MUT. BEN. ASSOCIATION,
AUSTIN, Texas, July 1, 1889.

Received of Dr. W. A. Morris, President, and Dr. F. E. Daniel, Sec. and Tr., P. M. B. A. of Texas, fifty nine dollars, net proceeds of assessment No. 12, for benefit ot certificate No. 68, held by the late Dr. J. M. Ross, of Brenham.

MRS. J. M. ROSS.

BRENHAM, Texas, July 3, 1889.

And this is the last of the Physicians' Mutual Benefit Association of Texas that will ever appear in the JOURNAL; but we trust the twelve ladies who have been the beneficiaries, even though the amount be small, will ever remember it, and take the will for the deed. We did our best.

